# Apparent weight loss and surface modification of household waste-derived mixed PE/PP microplastics by *Bacillus pumilus* and fungal isolates

**DOI:** 10.3389/fmicb.2026.1926623

**Published:** 2026-09-18

**Authors:** R. Rithika, Prithviraj Karak, P. Saranraj, K. Gayathri, M. Manigandan, Sayandeep K. Das, Afsona Parveen

**Affiliations:** 1 PG and Research Department of Microbiology, Sacred Heart College (Autonomous), Tirupattur, India; 2 Department of Physiology, Bankura Christian College, Bankura, India; 3 Department of Pathology, Shri B. M. Patil Medical College Hospital and Research Centre, BLDE (Deemed to be University), Vijayapura, India; 4 School of Allied and Healthcare Sciences, Centurion University of Technology and Management, Jatni, Khurda Road, Odisha, China

**Keywords:** apparent weight loss, *Aspergillus niger*, *Bacillus pumilus*, bioformulation, microplastics, polymer surface modification, *Trichoderma harzianum*

## Abstract

Microplastics are persistent contaminants of terrestrial and aquatic environments, creating a need for carefully validated remediation approaches. This laboratory study examined apparent weight loss and surface modification of household waste-derived mixed polyethylene/polypropylene (PE/PP) microplastics by *Bacillus pumilus*, *Aspergillus niger*, and *Trichoderma harzianum*. The isolates were incubated separately in carbon-deficient mineral salt medium containing microplastics as the only added carbon source. Apparent gravimetric weight loss, microbial growth, and pH were assessed, and *Bacillus pumilus*-treated particles were further examined by scanning electron microscopy (SEM), Fourier-transform infrared spectroscopy (FTIR), and X-ray diffraction (XRD). *Bacillus pumilus* produced the numerically greatest apparent weight loss (40.0% ± 1.1%), followed by *Aspergillus niger* (30.0% ± 1.0%) and *Trichoderma harzianum* (20.0% ± 1.0%); however, the overall comparison was not statistically significant (one-way ANOVA, *p* = 0.243). SEM demonstrated surface roughening, pits, and cracks, while XRD showed lower relative crystallinity in treated particles (48% ± 3%) than in the untreated control (65% ± 2%). FTIR demonstrated spectral changes consistent with oxidative surface modification. The bacterial isolate was placed within the *Bacillus pumilus* group by 16S rRNA analysis, and preliminary liquid and calcium-alginate formulations were prepared. Because the substrate was a mixed PE/PP fraction and the analytical endpoints did not directly measure mineralization or molecular-weight reduction, the findings are interpreted as laboratory-scale apparent weight loss and polymer-associated surface and structural modification rather than complete biodegradation.

## Graphical Abstract

**Figure:**
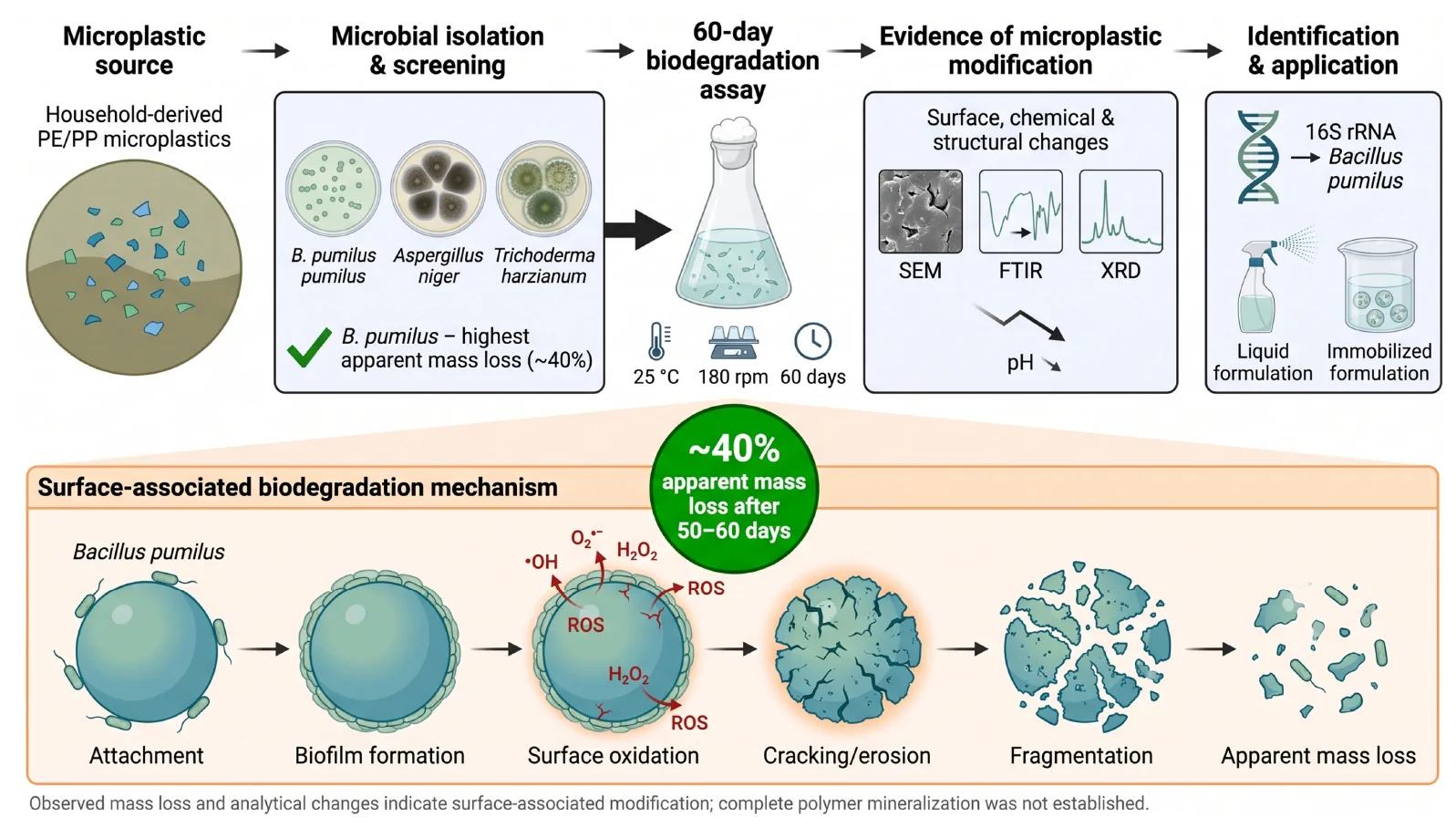
Overview of the isolation and screening of microplastic-associated microorganisms, 60-day incubation of household-derived mixed PE/PP particles, complementary SEM, XRD, FTIR, pH, and 16S rRNA analyses, and preliminary Bacillus pumilus formulation. The reported mass changes are apparent weight loss, and the analytical findings indicate surface-associated morphological, chemical, and relative structural modification; they do not establish polymer mineralization or complete biodegradation.

## 1 Introduction

Microplastics are synthetic polymer particles smaller than 5 mm and are now recognized as widespread environmental contaminants (Khalid et al., 2021; Wilkes and Aristilde, 2017). They are commonly classified as primary microplastics, which are manufactured at microscopic dimensions, or secondary microplastics, which arise through fragmentation of larger plastic products during physical, chemical, and biological weathering (Dong et al., 2021; Suardy et al., 2020). Their small size, persistence, and mobility promote dispersal across terrestrial, freshwater, marine, and atmospheric environments.

The global burden of plastic pollution continues to increase because only a limited proportion of discarded plastic is effectively recycled, while substantial quantities enter landfills or the wider environment (Garcia and Robertson, 2017). Once released, plastics weather and fragment progressively. Their persistence is governed by polymer type, molecular weight, crystallinity, hydrophobicity, additives, and prior weathering (Thakur et al., 2023), making conventional removal of dispersed microplastics technically difficult.

Microplastics can accumulate in soil, freshwater, marine systems, and wastewater-affected environments. Their surfaces may sorb metals, persistent organic contaminants, antibiotics, and other xenobiotics and can support complex microbial biofilms (Talbot et al., 2022; Tang et al., 2021). Experimental studies have associated microplastic exposure with oxidative stress, inflammation, immune disturbance, and adverse ecological effects, although risk varies with particle characteristics, concentration, exposure conditions, and the receiving ecosystem (Tang et al., 2021).

Their persistence, long-range transport, trophic transfer, and continuous environmental input make microplastics an important ecological concern (Ganguly et al., 2026; Jiao et al., 2026). Existing approaches–including filtration, separation, oxidation, landfilling, and incineration–can be energy-intensive, costly, ineffective for dispersed particles, or capable of generating secondary waste. Consequently, microbial and enzymatic strategies are being investigated as complementary approaches rather than established field-scale solutions (Jiao et al., 2026; Nadekar and Fulke, 2026). *Bacillus*, *Pseudomonas*, *Rhodococcus*, *Aspergillus*, and *Trichoderma* species have been associated with polymer colonization, biofilm formation, and surface modification, but reported effects vary widely with polymer composition, pretreatment, incubation conditions, microbial strain, and analytical endpoint (Barwant et al., 2026; Ganguly et al., 2026; Jiao et al., 2026; Nadekar and Fulke, 2026).

Recent international research has combined microbial screening with metagenomics, enzyme engineering, synthetic biology, multi-omics, and circular-bioeconomy approaches (Ganguly et al., 2026; Nadekar and Fulke, 2026). Nevertheless, complete mineralization of conventional PE and PP under environmentally realistic conditions remains uncommon. Many studies demonstrate mass loss, surface oxidation, altered crystallinity, or fragmentation without directly establishing conversion of polymer carbon to CO2 or biomass. Clear distinction among biodeterioration, fragmentation, depolymerization, assimilation, and mineralization is therefore essential.

Microbial colonization of polymer surfaces generally begins with adhesion and biofilm formation. Extracellular polymeric substances can improve attachment to hydrophobic surfaces and create a localized microenvironment for biochemical activity (Othman et al., 2021; Rogers et al., 2020). Enzymes and oxidative metabolites may subsequently alter susceptible surface regions; however, the responsible pathways depend strongly on the polymer and must be demonstrated experimentally rather than inferred from growth alone.

Bacterial and fungal genera investigated in this context include *Bacillus*, *Pseudomonas*, *Rhodococcus*, *Flavobacterium*, *Aspergillus*, and *Trichoderma* (Brunner et al., 2018; Chandra and Enespa, 2020; Rogers et al., 2020; Urbanek et al., 2018). *Bacillus* species are of interest because of their environmental adaptability, biofilm-forming capacity, and extracellular enzyme production (Auta et al., 2018; Sangeetha Devi et al., 2020). *Aspergillus niger* and *Trichoderma harzianum* can colonize complex substrates and have been investigated for polyethylene-associated surface modification (Chandra and Enespa, 2020; Sowmya et al., 2014).

Gravimetric mass loss is frequently used as a screening endpoint, but it cannot by itself distinguish polymer-chain degradation from loss of additives, loosely attached material, residual biomass, or unrecovered fragments. Interpretation is strengthened when gravimetry is combined with complementary techniques such as SEM, FTIR, XRD, molecular-weight analysis, metabolite identification, and mineralization assays (Howard et al., 2023; Thakur et al., 2023).

Accordingly, laboratory screening should use cautious terminology and clearly report the polymer composition, pretreatment, recovery procedure, controls, replicate structure, and analytical limitations. Surface damage or spectral change may demonstrate polymer-associated modification, but neither endpoint alone proves complete biodegradation.

The present study therefore evaluated apparent weight loss and surface modification of mixed household-derived PE/PP microplastics after incubation with *Bacillus pumilus*, *Aspergillus niger*, and *Trichoderma harzianum*. Gravimetric assessment, growth monitoring, pH measurement, SEM, FTIR, and XRD were used as complementary screening endpoints. A preliminary liquid inoculant and calcium-alginate bead preparation was also documented as a formulation-feasibility exercise without claiming enhanced degradation or field applicability.

## 2 Materials and methods

### 2.1 Study design and experimental overview

This laboratory-based experimental study isolated microorganisms from household waste-associated microplastic samples and screened their interaction with household-derived mixed PE/PP particles. Microplastics were enriched in carbon-deficient mineral salt medium (MSM), where they served as the only added carbon source (Howard et al., 2023).

Selected isolates were evaluated by apparent gravimetric weight loss, growth assessment, pH monitoring, SEM, FTIR, and XRD. The bacterial isolate showing the greatest numerical apparent weight loss was further characterized by 16S rRNA gene sequencing and used for preliminary formulation preparation.

### 2.2 Collection of microplastic samples

Microplastic samples colonized by naturally occurring microbial communities were collected from household waste dump soil in Tirupattur District, Tamil Nadu, India. The samples were collected from areas where domestic plastic waste had accumulated and showed visible contact with soil. The collected samples were transferred to sterile containers and transported to the laboratory for further processing.

Sampling was conducted during January 2025 at three household waste dump sites in Tirupattur District, Tamil Nadu, India. The sampled area was centered approximately at 12.495° N, 78.567°E. Fifteen composite samples were collected, five per site. Each composite comprised five subsamples collected from separate points within that site, pooled and homogenized in a sterile container. Samples were transported to the laboratory within 2 h in sterile single-use polypropylene containers or polyethylene sampling bags.

### 2.3 Pretreatment of microplastic samples

Collected microplastic particles were sonicated in an ultrasonic bath (40 kHz, 120 W) for 30 min to remove loosely attached debris and then treated with 10% hydrogen peroxide for 24 h at 100 mL per gram of microplastic. After repeated washing with sterile distilled water, the particles were used for enrichment in MSM. An uninoculated microplastic control subjected to the same H_2_O_2_ pretreatment and incubation conditions was maintained to assess abiotic mass loss and surface oxidation (Lee et al., 2023; Maw et al., 2022). The control showed an apparent weight loss of 2.1% ± 0.4%.

The small apparent mass change in the abiotic control was considered when interpreting the microbial treatments. SEM, FTIR, and XRD findings were therefore treated as complementary evidence of surface and structural modification rather than independent proof of polymer mineralization.

### 2.4 Characterization and preparation of microplastic particles

The experimental material consisted of mixed household-derived microplastics predominantly comprising PE and PP fractions, as indicated by FTIR. The particles were 25–100 μm and were used at 500 mg/L for growth studies (Bertoldi et al., 2025). Because the PE:PP ratio was not quantified, all findings represent the overall response of the mixed polymer fraction and cannot identify preferential modification of PE or PP.

Particle size was confirmed by standard sieve analysis followed by microscopic examination. Before inoculation, the particles were washed with 70% ethanol and exposed to ultraviolet light for 30 min on each side (Vural et al., 2025).

### 2.5 Preparation of mineral salt medium (MSM)

Mineral salt medium (1 L) lacking added carbon sources was prepared with KH_2_PO_4_ (0.5 g), (NH_4_)_2_SO_4_ (2.5 g), MgSO_4_⋅7H_2_O (0.5 g), CoCl_2_ (0.005 g), CaCl_2_ (0.001 g), Fe_2_(SO_4_)_3_ (0.01 g), Na_2_HPO_4_ (1 g), MnSO_4_⋅7H_2_O (0.001 g), (NH_4_)_6_Mo_7_O_24_ (0.00003 g), and distilled water. The medium was autoclaved (121 °C, 15 psi, 15 min), cooled, and supplemented with pretreated microplastic particles as the only added carbon source. Final pH before inoculation was 7.0 ± 0.2 (Rithika et al., 2025).

### 2.6 Enrichment and isolation of microplastic-associated microorganisms

Pretreated microplastic particles were inoculated into MSM and incubated under shaking conditions to enrich microorganisms capable of growing in the presence of microplastics as the only added carbon source. The enrichment cultures were incubated at 25 °C and 180 rpm for 60 days. Biofilm formation and visible microbial growth were monitored periodically during incubation.

After enrichment, microbial isolates were obtained by streak plating. Bacterial colonies were isolated on nutrient agar, while fungal colonies were isolated on Sabouraud’s dextrose agar. Two bacterial colonies and three fungal colonies were initially isolated. Based on growth and screening performance, one bacterial isolate and two fungal isolates were selected for further analysis (Salinas et al., 2023).

### 2.7 Morphological and biochemical characterization of microbial isolates

Bacterial isolates were examined for colony morphology, Gram staining, and biochemical characteristics (Catalase test, oxidase test, citrate utilization test, indole test, methyl red test, Voges–Proskauer test, starch hydrolysis test, urease test, and carbohydrate fermentation tests). The selected bacterial isolate showed circular, smooth colonies and appeared as Gram-positive rods. Biochemical tests included catalase and oxidase testing. Incubation conditions for bacterial culture were 37 °C for 24–48 h.

Fungal isolates were examined based on colony color, colony texture, and microscopic morphology using slide culture. The fungal isolates were identified morphologically as *Aspergillus niger* and *Trichoderma harzianum*. Incubation conditions for fungal culture were 28 °C for 5–7 days (Irawati et al., 2025).

### 2.8 Screening of selected isolates for apparent microplastic weight loss

*Bacillus pumilus*, *Aspergillus niger*, and *Trichoderma harzianum* were incubated separately with mixed PE/PP microplastics in MSM for 60 days at 25 °C and 180 rpm. The starting inocula were approximately 1 × 10^8^ CFU/mL for *Bacillus pumilus* and 1 × 10^6^ spores/mL for each fungal isolate (Liu B. et al., 2022; West et al., 2026). After incubation, residual particles were recovered, washed, dried, and assessed gravimetrically.

### 2.9 Gravimetric assessment of apparent weight loss

Residual particles were treated with sodium dodecyl sulfate for 2 h to reduce attached biomass, washed thoroughly with distilled water, oven-dried at 60 °C overnight, and weighed using an analytical balance with 0.02 mg readability (Irawati et al., 2025). Apparent weight loss was calculated as: apparent weight loss (%) = [(W_i_ − Wf)/W_i_] × 100.

W_i_ represents the initial dry mass and Wf the recovered dry mass after incubation. Because very small fragments may be incompletely recovered during gravimetric processing, recovered-mass differences were interpreted cautiously and were not equated with chain scission or mineralization.

### 2.10 Growth assessment and CFU quantification

Growth studies were conducted in triplicate in 200-mL flasks containing 500 mg/L mixed PE/PP particles. Cultures were incubated at 25 °C and 130 rpm for 30 days. OD_620_ and CFU/mL were recorded for *Bacillus pumilus* at days 0, 10, 20, and 30. OD was used as a liquid-culture biomass proxy for the fungal isolates, and colony radial expansion was recorded on solid medium. Because these endpoints differ biologically, growth measurements were summarized descriptively rather than treated as directly equivalent measures across bacteria and fungi (Hossain et al., 2024).

### 2.11 pH monitoring during incubation

The pH of the culture medium was monitored during microbial incubation with microplastics. pH readings (Eutech pH 700 digital pH meter) were recorded at baseline and at 15-days intervals up to day 60. The recorded time points were day 0, day 15, day 30, day 45, and day 60. The pH changes were compared among *Bacillus pumilus*, *Aspergillus niger*, and *Trichoderma harzianum* to assess metabolic activity during incubation (Chandramouli Swamy et al., 2024).

### 2.12 Scanning electron microscopy analysis

Scanning electron microscopy (JEOL JSM-6390 scanning electron microscope) was performed to assess surface morphological changes in microplastic particles after microbial treatment. After incubation, microplastic particles were washed twice with sterile distilled water, treated with SDS solution to remove biofilm, and then fixed using 2.5% glutaraldehyde for 2 h at 4 °C. Samples were dehydrated through a graded ethanol series (50%, 70%, 90%, 100%), air-dried overnight, and mounted on aluminum stubs using double-sided carbon tape. Samples were sputter-coated with gold (approximately 10 nm thickness) using a Quorum SC7620 sputter coater before imaging.

Scanning electron microscopy imaging was performed at the magnifications indicated on each micrograph. Representative fields were selected from three regions of the untreated control and *Bacillus pumilus*-treated samples. Imaging was performed at 10–30 kV in secondary-electron mode, and surface roughness, pits, cracks, cavities, deposits, and particle fragmentation were evaluated. These features were interpreted as surface morphological alteration rather than direct evidence of chain scission or mineralization (Wang et al., 2017; Yousafzai et al., 2025).

### 2.13 X-ray diffraction analysis

X-ray diffraction (PANalytical X’Pert PRO) was used to compare the relative crystalline and amorphous contributions of untreated and *Bacillus pumilus*-treated mixed PE/PP particles (Moura et al., 2023). Background correction, peak fitting, and area integration were performed in OriginPro using the same processing workflow for both groups. Relative crystallinity was calculated as CI (%) = Ac/(Ac + Aa) × 100.

Ac represents the integrated area beneath the resolved crystalline peaks, and Aa represents the integrated area of the diffuse amorphous halo. XRD changes were interpreted together with SEM and FTIR findings and not as an independent measure of mineralization.

### 2.14 Fourier-transform infrared spectroscopy analysis

Fourier-transform infrared spectroscopy spectra were acquired using a Shimadzu IRTracer-100 over 400–4000 cm^–1^ at 4 cm^–1^ resolution. Spectra were processed in Shimadzu LabSolutions and OriginPro. The same baseline-correction procedure was applied to untreated and *Bacillus pumilus*-treated samples to minimize background contributions, after which spectra were normalized to the C–H bending band near 1465 cm^–1^. Changes in hydroxyl, carbonyl, C–H, and C–O regions were assessed. The carbonyl index (CI) was calculated from baseline-corrected absorbance values as CI = A_1715_/A_1465_.


$$C\,I=A_{1715}/A_{1465}$$

A_1715_ represents absorbance in the carbonyl region and A_1465_ the reference C–H bending-band absorbance. Identical preprocessing, normalization, and calculation procedures were applied to the untreated control and *Bacillus pumilus*-treated samples.

### 2.15 DNA isolation and bacterial molecular identification

Genomic DNA was extracted from the selected bacterial isolate using a spin-column method after lysozyme and Proteinase K treatment. The bacterial 16S rRNA gene was amplified with universal primers 27F and 1492R, and the amplicon was sequenced by Sanger sequencing.

The resulting partial 16S rRNA sequence was compared with reference sequences in NCBI for taxonomic placement. The fungal isolates were identified by colony and microscopic morphology; a fungal molecular phylogeny was therefore not presented.

### 2.16 Phylogenetic analysis

The bacterial 16S rRNA sequence was compared with closely related reference and type-strain sequences. A Neighbor-Joining tree was constructed in MEGA 11 using the Kimura two-parameter model. Branch support was assessed with 500 bootstrap replicates, and *Paenibacillus polymyxa* ATCC 842^T^ was used as the outgroup. The study isolate is shown as *Bacillus pumilus* TPTD-1 (GenBank accession PZ803320).

### 2.17 Preliminary preparation of *Bacillus pumilus* formulations

Because *Bacillus pumilus* produced the greatest numerical apparent weight loss, it was used to prepare a liquid inoculant and calcium-alginate bead formulation as preliminary proof-of-concept preparations (Agake et al., 2025). No comparison of apparent weight loss was made between formulated and free cells.

#### 2.17.1 Liquid bioinoculant

The liquid preparation contained *Bacillus pumilus* at approximately 108 CFU/mL with 10% glycerol, 0.1% xanthan gum, and 0.5% yeast extract (Benítez et al., 2024).

#### 2.17.2 Immobilized bead formulation

Calcium-alginate beads containing *Bacillus pumilus* were prepared at approximately 3–5 mm diameter, with 2% activated charcoal incorporated into the matrix (Agake et al., 2025; Heidari et al., 2016). The formulation component was limited to documenting preparation feasibility.

### 2.18 Statistical analysis

Results are reported as mean ± standard deviation where applicable. Apparent weight-loss values from the three isolates were compared by one-way ANOVA with *p* < 0.05 as the significance threshold. The omnibus comparison was not statistically significant [*F*(2,6) = 1.81, *p* = 0.243]; consequently, no pairwise significance labels were assigned. The selection and interpretation of post hoc procedures followed Agbangba et al., 2024. Growth and pH patterns were summarized descriptively. XRD values are reported as mean ± SD for untreated and treated samples and interpreted as relative structural measurements.

### 2.19 Ethical and biosafety considerations

The study involved environmental microbial isolates obtained from household waste-associated soil and did not involve human participants, animals, or clinical specimens. The study was conducted according to institutional biosafety [Biosafety Level 1 (BSL-1) laboratory practices] and laboratory safety guidelines; separate ethical approval was not required for environmental microbial isolates.

## 3 Results

### 3.1 Isolation and screening of microplastic-associated microorganisms

Microplastic-associated microorganisms were isolated from household waste dump soil samples collected from Tirupattur District, Tamil Nadu, India. The collected microplastic particles were enriched in carbon-deficient mineral salt medium containing microplastics as the only added carbon source. After incubation, visible microbial growth and biofilm-like colonization were observed on the microplastic particles.

Based on colony morphology, microscopic examination, and subsequent bacterial molecular identification, one bacterial isolate was identified as *Bacillus pumilus*. Two fungal isolates were identified morphologically as *Aspergillus niger* and *Trichoderma harzianum*. These three isolates were selected for comparative assessment of apparent microplastic weight loss and surface-associated modification.

### 3.2 Gravimetric assessment of apparent microplastic weight loss

After incubation, particles were treated with SDS, washed, dried, and weighed. Each treatment comprised three biological replicates beginning with 100 mg of mixed PE/PP particles. The mean recovered mass and corresponding apparent weight loss are summarized in Table 1.

**TABLE 1:** Apparent gravimetric weight loss of mixed PE/PP microplastics after microbial treatment.

Microorganism	Initial weight	Final recovered weight (mean, mg)	Apparent weight loss (%)
*Bacillus pumilus*	100 mg	60	40.0 ± 1.1
*Aspergillus niger*	100 mg	70	30.0 ± 1.0
*Trichoderma harzianum*	100 mg	80	20.0 ± 1.0

Values are mean ± SD for apparent weight loss (*n* = 3). The overall comparison was not statistically significant (one-way ANOVA, [*F*(2,6) = 1.81, *p* = 0.243]; values are therefore interpreted descriptively.

*Bacillus pumilus* produced the greatest numerical apparent weight loss (40.0% ± 1.1%), followed by *Aspergillus niger* (30.0% ± 1.0%) and *Trichoderma harzianum* (20.0% ± 1.0%). The omnibus ANOVA did not demonstrate a statistically significant difference among isolates [*F*(2,6) = 1.81, *p* = 0.243]. *Bacillus pumilus* was selected for further surface and structural characterization because it produced the largest numerical change under the screening conditions. These values represent recovered-mass differences and should not be interpreted as proof of complete polymer biodegradation.

### 3.3 Growth characteristics of the selected isolates

OD_620_ was recorded as a liquid-culture biomass proxy, while colony radial expansion was recorded on solid medium. *Bacillus pumilus* showed an OD_620_ of 0.82 ± 0.15, *Aspergillus niger* 0.65 ± 0.12, and *Trichoderma harzianum* 0.48 ± 0.08. The reported colony-growth values were 3.7 ± 1.7, 3.3 ± 1.3, and 2.7 ± 0.5 mm/day, respectively (Table 2). Because bacterial and fungal growth measurements are not biologically interchangeable, these results were interpreted descriptively.

**TABLE 2:** Descriptive growth measurements and apparent weight loss of the selected isolates.

Microorganism	Reported colony growth (mm/day)	OD_620_	Apparent weight loss (%)
*B. pumilus*	3.7 ± 1.7	0.82 ± 0.15	40.0 ± 1.1
*A. niger*	3.3 ± 1.3	0.65 ± 0.12	30.0 ± 1.0
*T. harzianum*	2.7 ± 0.5	0.48 ± 0.08	20.0 ± 1.0

Values are mean ± SD (*n* = 3). Growth endpoints and apparent weight loss are presented descriptively; no superscript significance groups are assigned.

The growth measurements show that all three isolates remained metabolically active in microplastic-containing medium. They do not, by themselves, demonstrate assimilation of polymer carbon or explain the differences in recovered particle mass.

### 3.4 pH changes during incubation with mixed PE/PP microplastics

The culture pH was recorded at baseline and at 15-days intervals. *Bacillus pumilus* showed an initial rise from pH 7.0 to 7.5 by day 30, followed by a decline to 5.9 at day 60. *Aspergillus niger* and *Trichoderma harzianum* showed progressive acidification to pH 5.5 and 5.4, respectively (Tables 3, 4).

**TABLE 3:** Temporal pH changes during microbial incubation with mixed PE/PP microplastics.

Microorganism	Initial pH	Day 15	Day 30	Day 45	Day 60	Total Δ pH
*Bacillus pumilus*	7.0	7.3	7.5	6.5	5.9	−1.1
*Aspergillus niger*	7.0	7.2	6.7	5.7	5.5	−1.5
*Trichoderma harzianum*	7.0	7.0	6.6	5.6	5.4	−1.6

The recorded pH values are presented descriptively; no inferential significance groups are assigned.

**TABLE 4:** Descriptive pH patterns and apparent weight loss.

Parameter	*B. pumilus*	*A. niger*	*T. harzianum*
pH pattern	Biphasic	Progressive decrease	Progressive decrease
Final pH	5.9	5.5	5.4
Total Δ pH	−1.1	−1.5	−1.6
Apparent weight loss (%)	40.0%	30.0%	20.0%

The pH changes indicate metabolic activity during incubation. However, bulk-medium pH is influenced by multiple biological and chemical processes and cannot be used as a direct measure of polymer-carbon conversion.

Because the table reports central pH values without dispersion estimates at each time point, the pH trajectories are interpreted descriptively. Acidification is consistent with microbial metabolic activity but does not establish that polymer carbon was assimilated.

### 3.5 SEM assessment of microplastic surface morphology

Untreated particles showed comparatively compact surfaces, whereas *Bacillus pumilus*-treated particles displayed increased roughness, pits, cracks, cavities, and surface-associated deposits (Figure 1).

**FIGURE 1:**
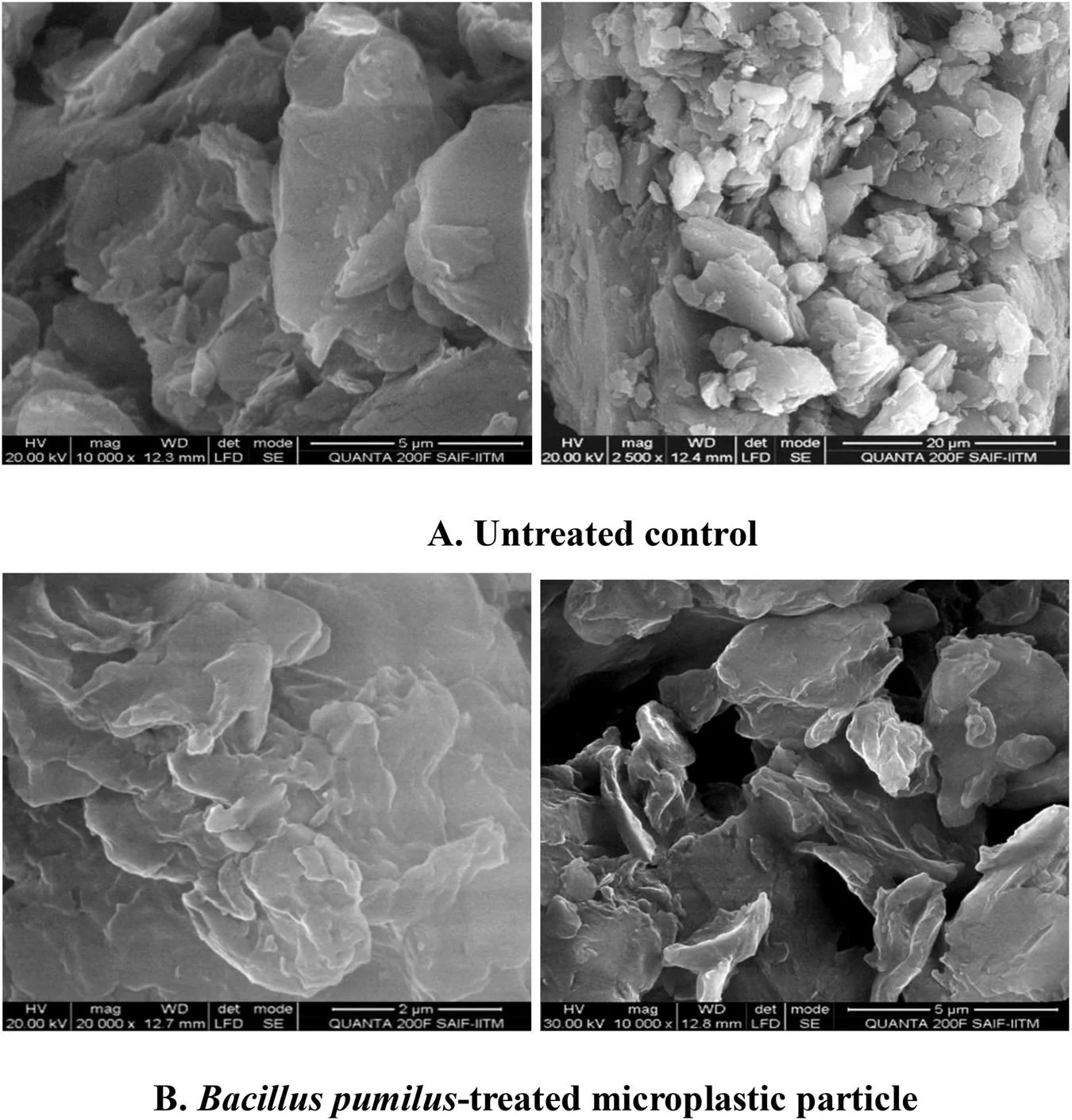
Representative SEM micrographs of household waste-derived mixed PE/PP particles before and after 60 days of incubation with *Bacillus pumilus* in carbon-deficient MSM at 25 °C and 180 rpm. **(A)** Untreated-control particles at 2,500× (left) and 10,000× (right). **(B)** *Bacillus pumilus*-treated particles at 10,000× (left) and 20,000× (right). Scale bars are 20 μm, 5 μm, 5 μm, and 2 μm, respectively. The observed roughness, pits, cracks, cavities, and deposits are interpreted as surface morphological alteration rather than proof of complete biodegradation.

These observations are consistent with microbial colonization and surface alteration. SEM alone cannot determine whether the changes reflect polymer-chain scission, loss of additives, fragmentation, or complete mineralization.

### 3.6 XRD assessment of relative structural changes

Untreated mixed PE/PP particles showed a semicrystalline diffraction pattern. *Bacillus pumilus*-treated particles showed reduced peak intensity and peak broadening. OriginPro peak fitting yielded relative crystallinity values of 65% ± 2% for the untreated control and 48% ± 3% for the treated group, with corresponding amorphous fractions of 35% ± 2% and 52% ± 3% (Figure 2 and Table 5).

**FIGURE 2:**
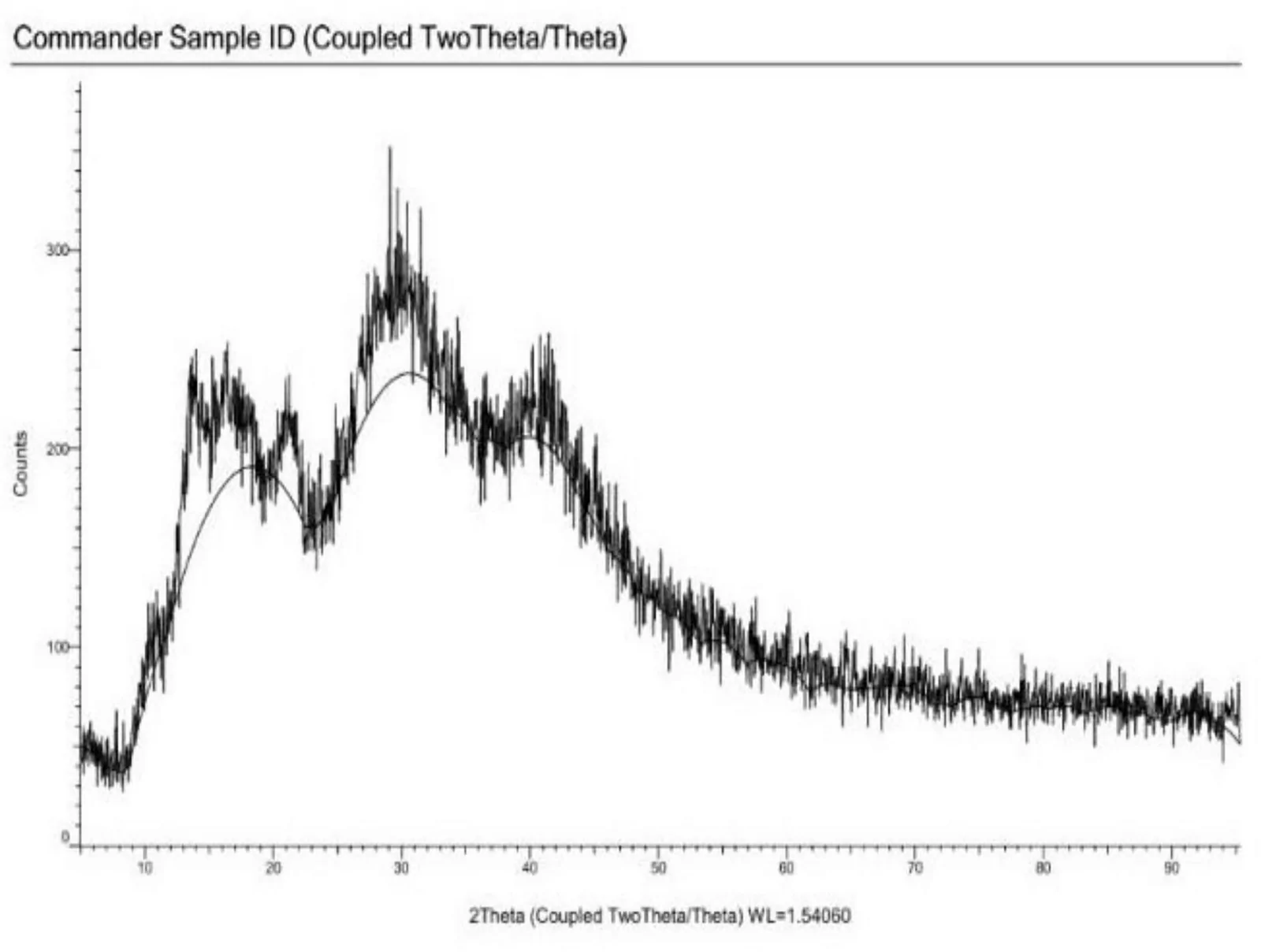
X-ray diffraction (XRD) patterns of untreated and *Bacillus pumilus*-treated household-derived mixed PE/PP particles. Peak broadening and lower intensity accompanied a decrease in relative crystallinity from 65% ± 2% to 48% ± 3% and an increase in the amorphous fraction from 35% ± 2% to 52% ± 3%. These results indicate relative structural modification of the mixed feedstock and are not presented as independent evidence of complete biodegradation.

**TABLE 5:** X-ray diffraction (XRD)-derived relative crystallinity of untreated and *Bacillus pumilus*-treated mixed PE/PP microplastics.

Parameter	Untreated control	*Bacillus pumilus*-treated
Crystallinity	65 ± 2	48 ± 3
Amorphous content	35 ± 2	52 ± 3

Values are mean ± SD (*n* = 3) and are interpreted as relative structural measurements derived using the same peak-fitting workflow for both groups.

The XRD findings indicate a difference in the relative crystalline and amorphous contributions after incubation with *Bacillus pumilus*. Because the feedstock was a heterogeneous mixed PE/PP fraction, these values do not identify which polymer was preferentially affected and do not independently establish mineralization.

Each group comprised three preparations, and results are summarized as mean ± SD. The same background-correction, peak-fitting, and area-integration procedure was applied to both groups.

### 3.7 FTIR assessment of surface chemical modification

Fourier-transform infrared spectroscopy spectra of the treated particles showed changes within regions associated with hydroxyl or unsaturated C–H stretching (3119.30 cm^–1^), carbonyl or C = C stretching (1633.01 cm^–1^), C–H bending (1398.64 cm^–1^), C–O stretching (1167.69 cm^–1^), and lower-wavenumber bending modes (841.26 and 617.15 cm^–1^) (Figure 3 and Table 6). Because the feedstock contained mixed PE and PP, the spectral changes represent the combined material and cannot identify preferential modification of either polymer.

**TABLE 6:** Fourier-transform infrared spectroscopy (FTIR) bands observed in *Bacillus pumilus*-treated mixed PE/PP microplastics.

Wave number	Possible functional group	Interpretation
3119.30 cm^–1^	−OH or =C–H stretching	Suggestive of hydroxylation or surface oxidation
1633.01 cm^–1^	C = O or C = C stretching	Suggestive of oxidative modification or chain alteration
1398.64 cm^–1^	C–H bending	Persistence of polymer backbone with possible modification
1167.69 cm^–1^	C–O stretching	Suggestive of oxidized surface groups
841.26 cm^–1^	Alkene/aromatic bending region	Possible structural modification
617.15 cm^–1^	C–H bending region	Possible chain alteration or surface weakening

After identical baseline correction and normalization, the carbonyl index increased from 0.08 ± 0.02 in the untreated control to 0.31 ± 0.04 in *Bacillus pumilus*-treated particles. This semiquantitative change is consistent with increased surface oxidation but does not establish polymer-chain scission or mineralization.

**FIGURE 3:**
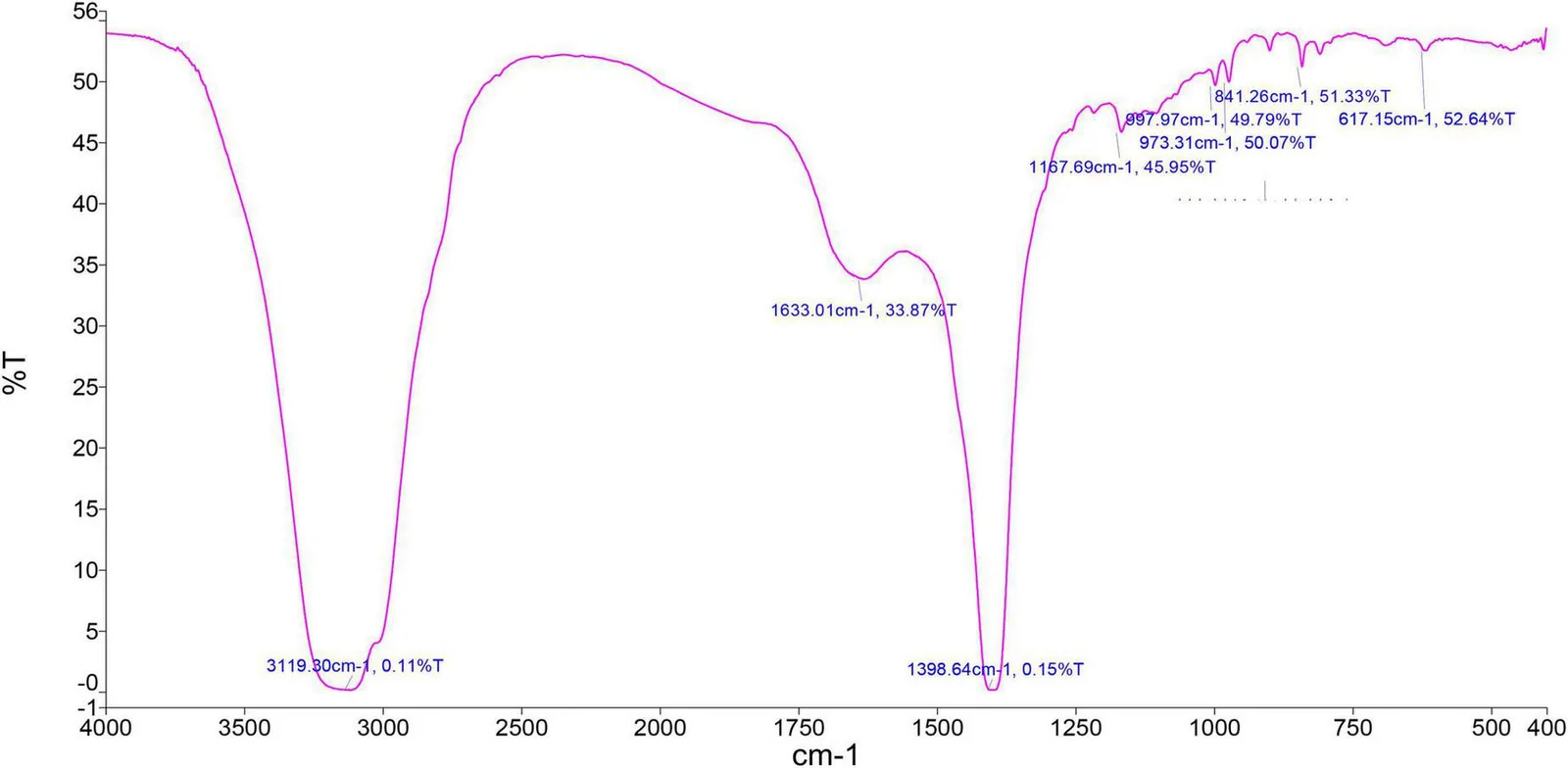
Fourier-transform infrared spectroscopy (FTIR) spectrum (400–4000 cm^−1^) of *Bacillus pumilus*-treated household-derived mixed PE/PP particles after 60 days of incubation in MSM.

The bands at 3119.30, 1633.01, and 1167.69 cm^–1^ and the increase in carbonyl index from 0.08 ± 0.02 to 0.31 ± 0.04 provide semiquantitative evidence of oxidative surface modification. The results are interpreted together with SEM and XRD findings.

Taken together, the SEM, FTIR, and XRD findings indicate surface-associated morphological, chemical, and relative structural changes in the mixed PE/PP material after bacterial incubation; they do not independently demonstrate complete biodegradation.

### 3.8 Molecular identification of the bacterial isolate

The bacterial isolate selected for further characterization was sequenced at the 16S rRNA locus and designated *Bacillus pumilus* TPTD-1. The sequence is reported under GenBank accession PZ803320. Phylogenetic analysis placed the isolate within the *Bacillus pumilus* group in association with reference strains, including *Bacillus pumilus* ATCC 7061^T^ (Figure 4).

**FIGURE 4:**
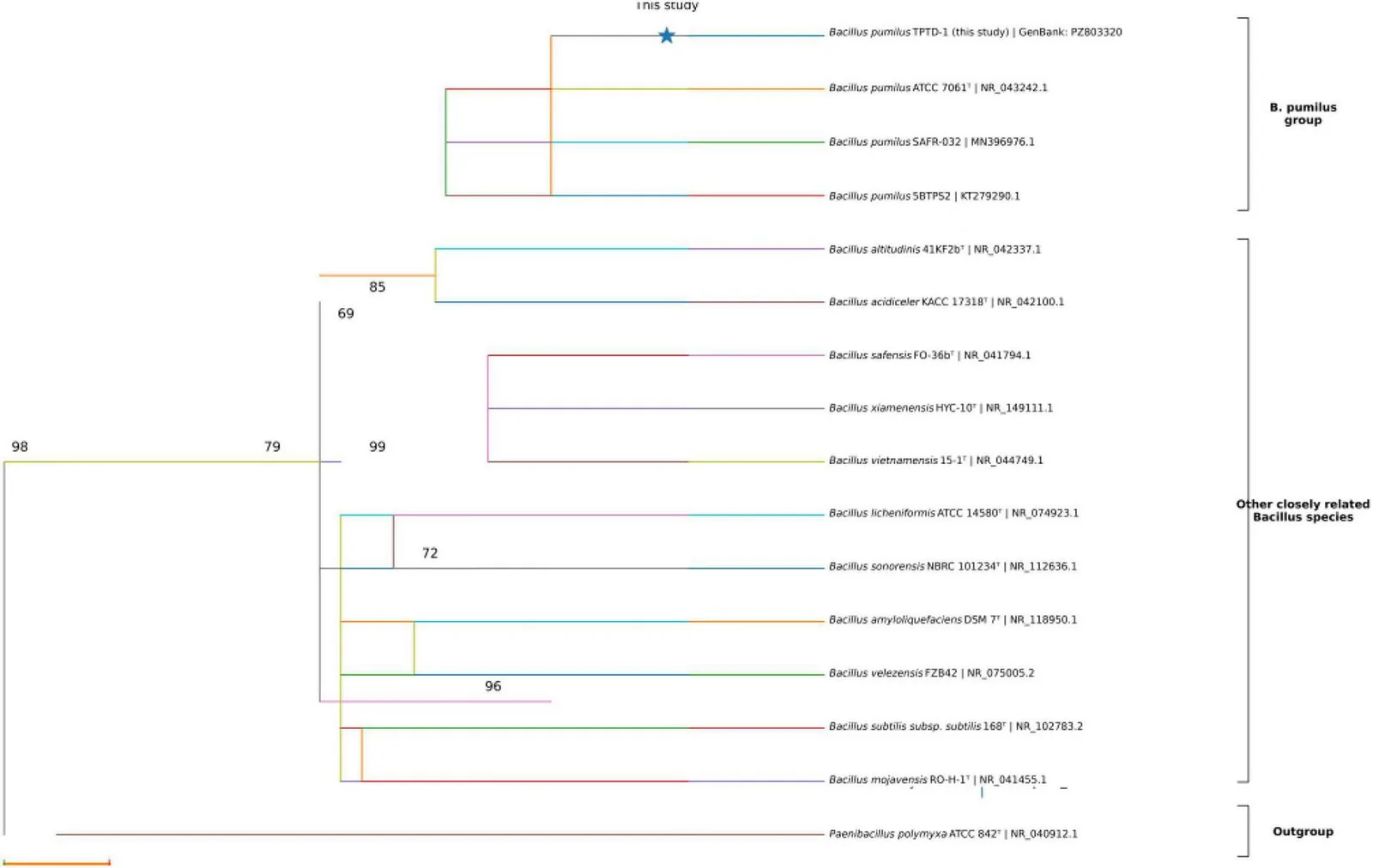
Neighbor-joining phylogenetic tree based on bacterial 16S rRNA sequences showing *Bacillus pumilus* TPTD-1 (PZ803320; this study), related *Bacillus* sequences, and the *Bacillus pumilus* ATCC 7061^T^ type strain. The tree was constructed in MEGA 11 using the Kimura two-parameter model. Bootstrap support from 500 replicates is shown at the nodes, and *Paenibacillus polymyxa* ATCC 842^T^ was used as the outgroup. The scale bar represents nucleotide substitutions per site. The blue star marks *Bacillus pumilus* TPTD-1 sequenced in the present study.

### 3.9 Preliminary *Bacillus pumilus* formulation preparation

A liquid inoculant and a calcium-alginate bead preparation were produced as proof-of-concept formulations after *Bacillus pumilus* showed the greatest numerical apparent weight loss in the isolate screen.

The liquid preparation contained approximately 10^8^ CFU/mL with glycerol, xanthan gum, and yeast extract. The calcium-alginate beads measured approximately 3–5 mm and incorporated activated charcoal. Representative preparations are shown in Supplementary Figure 1.

This component demonstrates only that the two formulation formats could be prepared. It does not demonstrate improved apparent weight loss, prolonged shelf stability, release kinetics, environmental performance, or field efficacy.

## 4 Discussion

This study screened microorganisms isolated from household waste-associated soil for their interaction with mixed PE/PP microplastics under controlled laboratory conditions. *Bacillus pumilus* produced the largest numerical apparent weight loss, while SEM, FTIR, and XRD demonstrated surface-associated morphological, chemical, and relative structural differences. These endpoints support polymer-associated modification but do not establish complete biodegradation or mineralization.

Microbial effects on plastics depend on polymer composition, molecular weight, crystallinity, additives, surface hydrophobicity, particle size, pretreatment, culture conditions, and microbial physiology (Ganguly et al., 2026; Thakur et al., 2023). The carbon-deficient MSM used here favored organisms able to persist in close association with the particles, but growth in this medium does not prove that polymer carbon was assimilated.

The numerical apparent weight-loss ranking was *Bacillus pumilus* (40.0% ± 1.1%), *Aspergillus niger* (30.0% ± 1.0%), and *Trichoderma harzianum* (20.0% ± 1.0%). The omnibus comparison was not statistically significant (*p* = 0.243), so the ranking is descriptive and no pairwise superiority is claimed.

Published values vary considerably because studies use different polymers, particle sizes, pretreatments, culture media, incubation periods, recovery methods, and analytical definitions. Direct numerical comparison must therefore be cautious and should distinguish recovered-mass change from mineralization.

The 40.0% ± 1.1% value for *Bacillus pumilus* represents apparent weight loss of a mixed PE/PP fraction under the specific laboratory conditions used here. It should not be interpreted as a polymer-specific transformation rate or extrapolated directly to field conditions (Rogers et al., 2020; Thakur et al., 2023).

For comparison, *Pseudomonas* sp. *ADL15* and *Rhodococcus* sp. *ADL36* produced reported PP mass losses of 17.3% and 7.3%, respectively, after 40 days (Habib et al., 2020). Other studies involving *Lysinibacillus* and *Bacillus* strains have reported polymer colonization and mass or structural changes under different experimental conditions (Jeon et al., 2021; Liu X. et al., 2022). These studies demonstrate the strong dependence of apparent mass-loss and modification endpoints on both strain and protocol.

Fungal studies show similar variability. Saira et al. (2022) reported an apparent LDPE mass loss of 22.9% with *Aspergillus niger* after 1 month, whereas Ahmed et al. (2025) reported 45.62% for *Aspergillus niger* and 36.01% for *Trichoderma harzianum* in liquid medium but substantially lower values in soil. These results are not directly equivalent to the present mixed-particle experiment but provide a contemporary context for the observed fungal responses.

The higher numerical response of *Bacillus pumilus* may reflect strain-specific attachment, biofilm formation, survival under nutrient limitation, or interaction with accessible components of the mixed feedstock. Because enzyme activity was not measured directly, the involvement of specific hydrolases or oxidoreductases remains a hypothesis for future testing (Auta et al., 2018; Danso et al., 2019; Sangeetha Devi et al., 2020; Sivan et al., 2006).

*Aspergillus niger* and *Trichoderma harzianum* showed lower numerical apparent weight loss than *Bacillus pumilus*. Fungal hyphae, extracellular metabolites, organic acids, and oxidative enzymes can contribute to polymer-surface modification (Ahmed et al., 2025; Álvarez-Barragán et al., 2016; Brunner et al., 2018; Sowmya et al., 2014), but the present study did not directly link a particular enzyme or metabolite to the observed mass change.

OD_620_, CFU measurements, and colony expansion confirmed microbial activity during incubation. However, these endpoints are not directly comparable across bacteria and filamentous fungi and cannot distinguish utilization of polymer carbon from growth supported by carryover nutrients, additives, or surface-associated organic material.

The pH increased in the *Bacillus pumilus* culture through day 30 and then declined, whereas both fungal cultures acidified progressively. These patterns are compatible with active metabolism and production of soluble metabolites. Nevertheless, bulk-medium pH is nonspecific and may not reflect conditions at the plastic–biofilm interface.

Scanning electron microscopy demonstrated greater roughness, pits, cracks, cavities, and deposits on treated particles than on the control. Such features are consistent with colonization and biodeterioration, but similar changes may also arise from pretreatment, mechanical damage, oxidation, or fragmentation. SEM therefore provides morphological support rather than proof of mineralization.

X-ray diffraction showed lower relative crystallinity and a larger amorphous contribution after *Bacillus pumilus* incubation. The difference may reflect alteration of accessible amorphous regions, disruption of ordered domains, or heterogeneity in the mixed particle fraction. XRD cannot identify mineralization and should be interpreted with complementary chemical and mass-balance data.

Fourier-transform infrared spectroscopy showed increased oxygen-associated spectral features and a higher carbonyl index after treatment. Because H_2_O_2_ pretreatment can oxidize polymer surfaces, the identically pretreated uninoculated control is important for interpretation. The smaller apparent mass change in that control supports an association with microbial incubation, while the analytical endpoints remain insufficient to distinguish chain scission from surface oxidation alone.

The bacterial 16S rRNA tree placed the isolate within the closely related *Bacillus pumilus* group and included the relevant type strain. Because closely related *Bacillus* taxa may be difficult to resolve using partial 16S rRNA sequences alone, additional housekeeping-gene or whole-genome analysis would provide stronger species-level confirmation. The fungal identifications were morphology-based; future studies should add ITS sequencing and type/reference-strain phylogenies (Manter and Vivanco, 2007).

The liquid and calcium-alginate preparations are preliminary formulation-feasibility demonstrations. They were not used to establish enhanced apparent weight loss, prolonged shelf stability, release behavior, scale-up performance, or environmental efficacy. These outcomes require dedicated comparative experiments before practical application can be proposed.

The environmental relevance of the findings is constrained by the controlled laboratory system, including defined temperature and agitation, carbon-deficient medium, relatively high particle concentration, and absence of natural microbial communities. Soil, sediment, freshwater, and marine environments additionally impose fluctuating moisture, oxygen, ultraviolet exposure, nutrients, organic matter, particle aggregation, competition, and contaminant mixtures.

Environmental microplastics also vary in polymer identity, crystallinity, additive composition, weathering history, and biofilm coverage. Consequently, the observed apparent weight loss should be treated as a screening result rather than an estimate of what would occur *in situ*. Environmentally realistic matrices, concentrations, and long-term exposure conditions are needed for translation.

The study therefore demonstrates association between microbial incubation and recovered-mass differences together with SEM, FTIR, and XRD changes. It does not demonstrate complete conversion of PE/PP carbon to CO2, biomass, or defined metabolites. Future validation should incorporate standardized respirometry, molecular-weight analysis, polymer-specific mass balance, metabolite profiling, and enzyme assays.

The principal limitations are the heterogeneous mixed PE/PP substrate, absence of a quantified PE:PP ratio, reliance on gravimetric recovery, possible loss of small fragments, potential influence of H_2_O_2_ pretreatment, absence of direct mineralization and molecular-weight endpoints, morphology-based fungal identification, and controlled laboratory conditions. These limitations prevent polymer-specific or field-scale conclusions.

Very small fragments may be incompletely captured by gravimetric recovery. Although SDS washing reduced attached biomass and the abiotic control showed only limited apparent weight loss, future studies should standardize filtration, recover all particle-size fractions, and use polymer-specific mass balance to improve gravimetric accuracy.

Future studies should test chemically characterized PE and PP separately under identical conditions; quantify CO2 evolution or oxygen consumption; measure molecular-weight distributions; characterize soluble and particulate products by GC–MS or LC–MS; assay candidate enzymes; sequence fungal ITS regions; compare free and formulated cells; and evaluate long-term performance in environmentally realistic microcosms.

## 5 Conclusion

*Bacillus pumilus* produced the greatest numerical apparent weight loss among the three screened isolates, while SEM, FTIR, and XRD demonstrated surface-associated morphological, chemical, and relative structural differences in treated mixed PE/PP particles. The isolate comparison was not statistically significant, and the mixed feedstock prevented polymer-specific attribution. Accordingly, the findings support laboratory-scale apparent weight loss and surface/structural modification, not complete biodegradation or mineralization. Separate-polymer experiments, direct mineralization and molecular-weight measurements, metabolite characterization, and environmentally realistic validation are required before remediation applications can be considered.

## Data Availability

The raw data supporting the conclusions of this article will be made available by the authors, without undue reservation. The partial 16s rRNA sequencing generated in this study is available in GenBank under accession number PZ803320.

## Supplementary material

Supplementary Figure 1 Representative preliminary Bacillus pumilus formulations: **(A)** liquid inoculant containing approximately 10^8^ CFU/mL with glycerol, xanthan gum, and yeast extract; **(B)** calcium-alginate beads approximately 3-5 mm in diameter containing activated charcoal. These images document preparation feasibility and do not demonstrate enhanced apparent weight loss, shelf stability, release kinetics, or field efficacy.
